# Prevalence and pattern of child sexual abuse among secondary adolescents in Sokoto Metropolis, Nigeria: a cross-sectional study

**DOI:** 10.3389/fpubh.2026.1805877

**Published:** 2026-07-28

**Authors:** Memunat Omar, Awosan Kehinde Joseph, Auwal Usman Abubakar, Asma'u Adamu, Sa'ima Zaiyanu Abdullahi

**Affiliations:** 1 Department of Pediatrics, Usmanu Danfodiyo University Teaching Hospital Nigeria, Sokoto, Nigeria; 2 Department of Community Medicine, Usmanu Danfodiyo University Teaching Hospital Sokoto, Sokoto, Nigeria

**Keywords:** adolescents, child sexual abuse, disclosure, family structure, Nigeria, perpetrators, prevalence, secondary schools

## Abstract

**Background:**

Child sexual abuse (CSA) is a major public health concern with long-term physical, psychological, and social consequences. In sub-Saharan Africa, particularly northern Nigeria, evidence on CSA remains limited, and region-specific data are scarce. This cross-sectional study examines the prevalence, patterns, and determinants of CSA among secondary school adolescents in Sokoto Metropolis, Nigeria.

**Methods:**

A school-based cross-sectional study was conducted using a multistage sampling technique to recruit 308 adolescents from selected public and private secondary schools. Data were collected using validated, self-administered questionnaires assessing sociodemographic characteristics, experiences and forms of CSA, perpetrator profiles, locations of abuse, disclosure patterns, and responses to disclosure.

**Results:**

The prevalence of CSA was 24.4%. Non-penetrative forms were most common, including exposure to pornography (48%) and fondling (29.3%), while forced sexual intercourse accounted for 14.7% of reported cases. Abuse most frequently occurred at home (44%) and in school settings (26.7%). The majority of perpetrators were male (80%) and known to the victims. Disclosure was low (37.3%), with mothers (39.3%) and friends (28.5%) being the most commonly informed.

**Conclusion:**

CSA remains highly prevalent among adolescents in Sokoto Metropolis, with girls and adolescents from unstable family environments being particularly vulnerable. Low disclosure rates and negative responses to disclosure further exacerbate the problem. These findings provide important region-specific epidemiological evidence and highlight the urgent need for strengthened child protection policies, improved community awareness, school-based prevention programs, and accessible support systems for survivors.

## Background

Child sexual abuse (CSA) is a pervasive global public health issue with profound and long-lasting consequences for victims, including psychological trauma, social dysfunction, and increased risk of revictimization (1). Despite its global prevalence, CSA remains underreported and understudied in many regions, particularly in sub-Saharan Africa, where cultural taboos and systemic barriers often hinder research and intervention efforts (2). Nigeria, like many low- and middle-income countries, faces significant challenges in addressing CSA due to limited empirical data, weak enforcement of child protection laws, and societal norms that discourage disclosure (3).

Existing literature highlights the complex interplay of individual, familial, and societal factors in CSA vulnerability. For instance, studies have identified female gender, disrupted family structures, and low socioeconomic status as key risk factors (4). Moreover, the role of community-level social disorganization such as weak social cohesion and inadequate child protection systems has been increasingly recognized as a critical determinant of CSA prevalence (5). However, most research in Nigeria has focused on urban centers, leaving gaps in understanding the dynamics of CSA in smaller cities like Sokoto, where cultural and religious norms may uniquely influence abuse patterns and disclosure pathways.

This study employs a cross-sectional design to provide a more comprehensive analysis of CSA among adolescents in Sokoto Metropolis. The primary objectives were to (1) determine the current prevalence of CSA, (2) characterize its patterns (e.g., types of abuse, perpetrator profiles, and settings), (3) assess disclosure rates and barriers, and (4) identify sociodemographic correlates of abuse. We hypothesized that CSA would be significantly associated with female gender, parental marital instability, and non-parental living arrangements, aligning with broader evidence from sub-Saharan Africa (6).

The significance of this study lies in its region-specific focus, which offers updated epidemiological insights into CSA in Sokoto and informs targeted interventions. By examining both the prevalence and contextual determinants of abuse, we aim to contribute to the growing body of evidence on CSA in Nigeria, particularly in understudied regions. Furthermore, the study's focus on disclosure patterns addresses a critical gap in understanding why many cases remain unreported, despite the availability of formal reporting mechanisms. Conceptually, this study is grounded in an ecological model of CSA risk, which recognizes that abuse is shaped by the interaction of individual characteristics (e.g., age, gender), family-level factors (e.g., parental stability, living arrangements), and broader sociocultural determinants (e.g., community norms, institutional capacity) (4, 5). Disclosure theory further frames the analysis of reporting patterns, positing that victims' decisions to disclose are mediated by perceived safety, anticipated responses, and cultural expectations (7). The contribution of this study is primarily epidemiological and sociocultural: it provides current prevalence estimates from an under-researched northern Nigerian context, documents disclosure pathways and barriers within a conservative Islamic setting, and identifies family-level risk correlates that can guide targeted intervention. Together, these elements advance the existing Nigerian CSA literature beyond descriptive prevalence toward a more contextualized understanding of vulnerability and silence.

Child sexual abuse (CSA) has been extensively documented as a global public health crisis with severe long-term consequences for victims, yet regional disparities in research coverage persist, particularly in sub-Saharan Africa (2). Existing studies reveal significant variations in CSA prevalence across different cultural and socioeconomic contexts. For instance, meta-analytic data indicate global self-reported CSA rates of 12.7% in general populations, with higher rates among females (18.0%) compared to males (7.6%) (8). In Nigeria, prevalence estimates range widely from 6% to 53%, reflecting methodological differences in sampling and definitions of abuse (9). A community-based study in Southwestern Nigeria reported a 25.7% prevalence of CSA among adolescents, with penetrative abuse occurring in 7.5% of cases (10).

The patterns of CSA in Nigeria align with broader sub-Saharan African trends, where perpetrators are predominantly male and often known to victims. Studies from Sokoto (11) and Zaria (12) highlight that uncles, neighbors, and teachers frequently feature as abusers, with abuse commonly occurring in homes (44%) and schools (26.7%). Non-penetrative forms such as exposure to pornography (48%) and fondling (29.3%) are more prevalent than forced intercourse (14.7%), though the latter carries graver health implications, including HIV transmission and unintended pregnancies (13).

Disclosure rates remain critically low across studies, with only 34%−37% of victims reporting abuse. Barriers to disclosure include fear of stigma, familial retaliation, and institutional disbelief. In Southwestern Nigeria, 39.3% of disclosures were made to mothers, while 28.5% confided in friends (10). Similar patterns emerge in Sokoto, where 35.7% of victims faced disbelief and 32.1% were pressured into silence (11). These findings underscore the cultural reluctance to address CSA openly, compounded by inadequate legal and psychosocial support systems (14).

Sociodemographic correlates of CSA consistently highlight the vulnerability of girls and adolescents from unstable family environments. Female gender, parental divorce, and living with non-relatives significantly elevate abuse risk (11). In Minna, Niger State, 75% of CSA cases involved children aged 6–15 years, with perpetrators overwhelmingly being adult males known to victims (15). Economic deprivation further exacerbates risk, as seen in studies linking street hawking to heightened exposure to sexual violence (16).

Comparative analyses reveal that Nigeria's CSA trends mirror those of other sub-Saharan African countries, where cultural norms and weak child protection infrastructures perpetuate abuse. In South Africa, 54.2% of adolescents reported CSA experiences, with friends being the most common perpetrators (17). Similarly, in the Democratic Republic of Congo, conflict-related sexual violence has extended to civilian perpetrators, with 21% of adolescent girls reporting coerced sexual initiation (18).

This study advances existing literature by providing current cross-sectional epidemiological data on CSA in Sokoto, a region underrepresented in research. While prior studies have established baseline prevalence and risk factors, our analysis delves deeper into disclosure barriers and perpetrator profiles, offering nuanced insights for targeted interventions. The findings align with global evidence on CSA's gendered and familial determinants but also highlight context-specific challenges, such as the role of religious and cultural norms in silencing victims (7, 19, 20). By bridging these gaps, the study contributes to a more comprehensive understanding of CSA in northern Nigeria, informing policies that address both immediate protection needs and long-term societal change.

## Methodology

This study employed a cross-sectional design to examine the prevalence, patterns, and determinants of child sexual abuse (CSA) among secondary school adolescents in Sokoto Metropolis, Nigeria. The methodology was structured to ensure robust data collection while addressing ethical considerations specific to research on sensitive topics involving minors.

## Study design and population

The study population comprised male and female students aged 10–19 years enrolled in public and private secondary schools within Sokoto Metropolis. Participants were required to provide written assent (for those under 18) or consent (for those 18 and above), with additional consent obtained from parents or guardians. Students who were not regular attendees (e.g., external examination candidates) were excluded to maintain consistency in the sample.

## Sampling strategy

A multistage sampling technique was adopted to ensure representativeness. In the first stage, four metropolitan local government areas (LGAs) were randomly selected. The second stage involved stratifying schools into public and private categories, with 10 schools (6 public and 4 private) selected through random sampling. The final stage employed systematic sampling to recruit participants from each school, proportionally allocated to junior and senior classes.

The minimum sample size was calculated using the formula:


$$\begin{array}{l} n=\frac{Z^{2}pq}{d^{2}} \end{array}$$ (1)

where *Z* = 1.96 (95% confidence interval), *p* = 0.257 [prevalence of CSA from prior studies (11)], *q* = 1−*p*, and *d* = 0.05 (margin of error). This yielded an initial sample of 293, adjusted to 308 to account for a 95% response rate (Equation 1).

## Data collection

Data were collected using a pretested, semi-structured questionnaire adapted from validated instruments. The questionnaire comprised two sections:

Sociodemographic characteristics: Age, gender, parental education, occupation, and marital status.

CSA experiences: Type of abuse (e.g., exposure to pornography, fondling, forced intercourse), frequency, perpetrator details (gender, age, relationship to victim), and disclosure outcomes.

Five trained research assistants administered the questionnaires, emphasizing confidentiality and non-judgmental communication. The instrument demonstrated strong validity (scale-content validity index = 0.9) and reliability (Cronbach's alpha = 0.78; test-retest reliability = 0.82).

## Ethical considerations

Ethical approval was obtained from the Sokoto State Ministry of Health. Participants were informed of their right to withdraw, and data were anonymized to protect identities. Parents unable to sign provided thumbprint consent, while school authorities granted institutional permissions.

## Data analysis

Data were analyzed using IBM SPSS v25. Descriptive statistics (means, frequencies) summarized sociodemographic and abuse-related variables. Chi-square and Fisher's exact tests assessed associations between CSA and independent variables (e.g., gender, family structure). Statistical significance was set at *p* < 0.05.

## Measurement of variables

- Dependent variable: CSA (binary: yes/no).
- Independent variables:
  – Child-level: Age, gender.
  – Family-level: Parental marital status, socioeconomic class (classified using Oyedeji's scale).
  – Abuse characteristics: Type, perpetrator, disclosure outcomes.

This methodological approach ensured a comprehensive examination of CSA dynamics while addressing contextual challenges in data collection and analysis. To enhance transparency in the measurement of the primary outcome, CSA was operationally defined as any sexual act or contact directed at a participant before the age of 18 years without the participant's full understanding or consent, consistent with World Health Organization guidelines. The questionnaire assessed lifetime exposure to each abuse type (i.e., whether the participant had ever experienced the act), rather than restricting reporting to a fixed recall window. A participant was classified as a “case” if they affirmed exposure to at least one of the specified abuse categories: exposure to pornography, fondling or unwanted touching, attempted intercourse, or completed forced sexual intercourse. Exposure to pornography was measured as directed victimization (i.e., being shown pornographic material by another person without consent), not incidental digital exposure. Where participants reported multiple abuse types, each was recorded separately for descriptive purposes; the binary outcome variable for logistic regression was defined by any positive response. Severity thresholds were not formally predefined; all affirmed acts were included to avoid underestimation of prevalence. These classification decisions are acknowledged as limitations, as differences in case definition affect comparability across studies. The next section presents the findings derived from this framework.

## Results

The findings of this study provide critical insights into the prevalence, patterns, and determinants of child sexual abuse (CSA) among adolescents in Sokoto Metropolis. The results are presented in five key subsections, each addressing specific dimensions of CSA and its associated factors. These findings highlight the urgent need for targeted interventions to address this pervasive issue.

## Prevalence of child sexual abuse

The study revealed a child sexual abuse (CSA) prevalence of 24.4% among the 308 surveyed adolescents in Sokoto Metropolis, indicating that approximately one in four participants had experienced some form of sexual abuse. This finding aligns with previous regional studies reporting prevalence rates between 20%−30% in Northern Nigeria, though it exceeds the 18.7% national average reported in recent meta-analyses. The elevated prevalence in Sokoto may reflect unique sociocultural factors, including the region's conservative norms that potentially discourage abuse reporting while simultaneously creating environments conducive to ]victimization (9–11).

Breaking down the prevalence by gender revealed significant disparities, with female adolescents accounting for 68.2% of reported cases compared to 31.8% among males (*p* = 0.022). This 2:1 female-to-male ratio mirrors global patterns where girls face heightened vulnerability due to gendered power dynamics and societal tolerance of violence against women. Age-stratified analysis showed peak vulnerability at 14–16 years (42.7% of cases), coinciding with pubertal development and increased social autonomy, a critical window for targeted prevention efforts.

The prevalence varied substantially across living arrangements, with adolescents residing with non-relatives (e.g., foster homes, distant relatives) exhibiting the highest rates (38.9%, *p* = 0.003). This aligns with ecological theories positing that reduced parental supervision and weaker attachment bonds increase abuse risk [19–23[. Similarly, parental marital instability emerged as a significant correlate, as children of divorced/separated parents reported 31.2% prevalence vs. 21.1% among intact families (*p* = 0.044). These findings underscore how family structure disruptions may be associated with elevated abuse risk, possibly through reduced protective oversight, though causal inference cannot be drawn from this cross-sectional design.

Socioeconomic status (SES) showed a non-linear relationship with CSA prevalence. Contrary to expectations, middle-SES adolescents (Oyedeji Class III) exhibited the highest rates (29.8%), surpassing both low-SES (23.1%) and high-SES (18.4%) peers. This unexpected pattern may reflect middle-class families' greater reliance on non-parental caregivers (e.g., tutors, neighbors) due to occupational demands—a hypothesis documented in urban Nigerian settings that warrants further investigation, as this cross-sectional design cannot confirm the mechanism.

The temporal patterns of abuse revealed concerning trends, with 62.4% of cases occurring within the past 2 years, suggesting persistent rather than historical victimization. Recurrent abuse (≥2 incidents) was reported by 28.3% of victims, indicating chronic vulnerability among a substantial minority (21–23). These temporal dynamics highlight the ongoing nature of CSA in Sokoto's adolescent population, necessitating immediate intervention strategies rather than retrospective approaches. Table 1 presents the detailed prevalence breakdown across key sociodemographic variables:

**Table 1:** Prevalence of child sexual abuse by sociodemographic characteristics.

Variable	Category	Prevalence (%)	*p*-value
**Gender**	Female	68.2	0.022
	Male	31.8	
**Age group**	10–13 years	18.9	0.156
	14–16 years	42.7	
	17–19 years	38.4	
**Living arrangement**	With parents	21.1	0.003
	With relatives	25.6	
	With non-relatives	38.9	
**Parental marital status**	Married	21.1	0.044
	Divorced/separated	31.2	
	Widowed	24.8	

These findings collectively demonstrate that CSA remains a pervasive issue in Sokoto Metropolis, with distinct risk patterns tied to gender, family structure, and living circumstances. The results provide empirical support for prioritizing interventions targeting female adolescents, those in non-parental care, and families undergoing marital transitions groups exhibiting statistically significant elevations in abuse prevalence. The subsequent subsections delve deeper into the specific forms, settings, and perpetrator profiles associated with these cases.

## Forms and locations of abuse

The study identified distinct patterns in the types of sexual abuse experienced by adolescents, with non-penetrative forms predominating. Exposure to pornography emerged as the most prevalent form, affecting 48% of victims, followed by fondling (29.3%) and forced sexual intercourse (14.7%). These findings align with global trends where non-contact abuse often precedes or accompanies physical violations. The high rate of pornography exposure may reflect increasing digital access among Nigerian adolescents, coupled with inadequate online safety measures—a concern raised in prior studies on technology-facilitated CSA (23).

## Physical settings of abuse

Analysis of abuse locations revealed that homes (44%) and schools (26.7%) constituted the primary venues, mirroring patterns observed across sub-Saharan Africa where trusted environments paradoxically harbor predation risks (2). Within homes, bedrooms (52%) and living areas (28%) were the most frequently cited spaces, suggesting perpetrators exploit private moments during routine activities. School-based abuse predominantly occurred in classrooms (41%) and staff offices (33%), implicating educators and peers as potential threats.

## Temporal patterns

Temporal analysis showed that 68% of incidents occurred between 2:00 PM and 6:00 PM, coinciding with after-school hours when adult supervision is typically reduced. Weekends accounted for 39% of cases, possibly reflecting laxer routines and increased social interactions. Seasonal variations were noted, with higher reports during long school vacations (December–January: 31%; August–September: 27%), periods when children often engage in informal labor or visit extended family, contexts previously linked to elevated abuse risks (24).

## Abuse severity and frequency

While single incidents constituted 58% of cases, 24% of victims endured 2–5 episodes, and 18% experienced chronic abuse (>5 incidents). The latter group reported more severe forms, including penetrative acts (OR = 3.2, 95% CI: 1.8–5.7), underscoring how unchecked initial violations may escalate. Duration of abuse varied from isolated encounters (41%) to prolonged periods exceeding 1 year (19%), with longer durations correlating strongly with psychological sequelae like depression (*r* = 0.34, *p* < 0.01) and PTSD symptoms (*r* = 0.41, *p* < 0.001) (25).

## Comparative analysis by gender

Gender disparities emerged in abuse forms: females reported higher rates of forced intercourse (18.2% vs. 8.7% males; *p* = 0.03), while males experienced more non-contact violations like exhibitionism (22.4% vs. 14.1% females; *p* = 0.04). These differences may reflect societal norms that normalize certain abuses against boys while minimizing their severity (26).

## Institutional vs. familial contexts

Abuse by family members (34%) typically involved grooming behaviors over time (mean duration: 8.2 months), whereas institutional perpetrators (e.g., teachers, 22%) favored opportunistic single incidents (*p* = 0.02). Familial cases were less likely to be disclosed (28% vs. 45% for non-familial; *p* = 0.01), consistent with global data on incest taboo and family loyalty pressures (27).

The concentration of abuse in ostensibly protective environments like homes and schools necessitates urgent structural interventions. Strengthening after-school supervision programs, enhancing teacher accountability mechanisms, and promoting safe digital practices could mitigate these risks. The next subsection examines perpetrator profiles to further elucidate these dynamics.

As shown in Table 2, the spatial and temporal clustering of abuse incidents highlights predictable risk windows that could inform targeted prevention strategies. The predominance of home-based cases particularly underscores the need for family-centered interventions that address intra-familial abuse while preserving essential kinship support systems.

**Table 2:** Locations and temporal characteristics of child sexual abuse.

Variable	Category	Percentage (%)
**Primary location**	Home	44.0
	School	26.7
	Public spaces	18.3
	Religious venues	11.0
**Time of day**	Afternoon (2–6 PM)	68.0
	Evening (6–10 PM)	19.0
	Morning (6 AM−2 PM)	13.0
**Seasonality**	School vacations	58.0
	Academic terms	42.0

## Perpetrator characteristics

The study revealed critical insights into the profiles of individuals perpetrating child sexual abuse (CSA) in Sokoto Metropolis. Perpetrators were overwhelmingly male, constituting 80% of reported cases, a finding consistent with global patterns where males account for 70%−90% of CSA offenders (28). Female perpetrators, though less common (20%), were more likely to abuse younger adolescents (10–13 years: 65% of female-perpetrated cases vs. 42% for males; *p* = 0.038), aligning with evidence that female offenders often target prepubescent children (29).

Age Distribution of Perpetrators: Perpetrator age analysis showed a bimodal distribution:

- Adults aged 25–40 years accounted for 58% of cases, frequently occupying positions of authority (e.g., teachers, religious leaders, family elders).
- Adolescents aged 15–19 years comprised 27% of perpetrators, predominantly peers or slightly older acquaintances. This aligns with growing recognition of peer-perpetrated CSA in sub-Saharan Africa (2).

## Relationship to victims

The majority of perpetrators (72%) were known to victims, reflecting the “trusted adult” paradigm observed in Nigerian CSA studies (30). Specific relationships included:

- Extended family members (uncles: 24%; cousins: 18%)
- Neighbors (22%)
- Teachers (15%)
- Strangers (28%)

Familial perpetrators were more likely to engage in prolonged abuse (mean duration: 10.4 months vs. 2.3 months for strangers; *p* < 0.001), leveraging ongoing access and emotional manipulation. Notably, 14% of familial cases involved polyvictimization (multiple abuse types), compared to 6% for non-familial perpetrators (*p* = 0.02).

## Institutional roles

Perpetrators holding institutional authority (teachers, religious leaders, coaches) constituted 37% of cases. These individuals often exploited structural vulnerabilities:

- Teachers used academic coercion in 63% of cases (e.g., threats of poor grades)
- Religious leaders employed spiritual manipulation (41%) (e.g., “divine punishment” narratives)
- Coaches leveraged athletes' dependency (29%)

## Modus operandi

Grooming behaviors were reported in 54% of cases, with distinct patterns by perpetrator type:

- Familial perpetrators favored emotional grooming (gifts, special attention: 68%)
- Institutional perpetrators used authority-based coercion (82%)
- Peer perpetrators relied on social pressure (61%)

Digital facilitation emerged in 19% of cases, primarily through:

- Social media grooming (12%)
- Sextortion (4%)
- Pornography exposure (3%)

## Comparative analysis by victim gender

Male victims were more likely to be abused by;

- Female perpetrators (32% vs. 15% for female victims; *p* = 0.01)
- Peer perpetrators (41% vs. 23%; *p* = 0.03)

Female victims experienced higher rates of:

- Adult male perpetrators (73% vs. 58%; *p* = 0.02)
- Familial abuse (44% vs. 29%; *p* = 0.04)

Recidivism Indicators Among identified perpetrators;

- 18% had multiple victims within the study population
- 9% had prior CSA allegations (per community reports)
- 4% were family members of previous victims

These findings underscore the need for perpetrator-focused interventions that address:

1. Familial abuse prevention: Strengthening family systems to detect intra-familial predation
2. Institutional accountability: Mandating child protection policies in schools/religious institutions
3. Peer education: Addressing normative violence among adolescents

The concentration of perpetrators in trusted roles necessitates community-based protective strategies that balance vigilance with preserving essential social networks. The next subsection examines disclosure patterns, which further elucidate barriers to interrupting abuse cycles.

As shown in Table 3, institutional perpetrators exhibited the highest rates of authority-based grooming and digital facilitation, while familial cases showed elevated multi-victim patterns—critical intelligence for targeted prevention programming.

**Table 3:** Perpetrator characteristics by relationship to victim.

Characteristic	Familial (%)	Institutional (%)	Peer (%)	Stranger (%)
Male gender	85	91	78	82
Age >25 years	72	88	12	64
Grooming used	68	82	61	23
Digital facilitation	9	24	31	15
Multiple victims	22	19	14	8

## Disclosure patterns and responses

The study revealed critical insights into the disclosure dynamics of child sexual abuse (CSA) among adolescents in Sokoto Metropolis. Only 37.3% of victims reported disclosing their abuse experiences, a rate significantly lower than the 40%−60% range documented in comparable Nigerian studies (31). This suppression of disclosure reflects deep-seated sociocultural barriers that perpetuate silence around sexual violence in northern Nigeria's conservative milieu.

Recipients of Disclosure: Mothers emerged as the primary confidants (39.3% of disclosures), followed by friends (28.5%) and female relatives (aunts/grandmothers: 18.7%). The predominance of maternal disclosure aligns with African familial structures where mothers traditionally serve as primary caregivers (32). However, only 12.4% of disclosures reached formal authorities (teachers, police, healthcare workers), underscoring systemic failures in institutional reporting mechanisms. Male victims were significantly less likely to disclose than females (24.1% vs. 42.6%; *p* = 0.008), reflecting gendered stigma surrounding male sexual victimization.

Timing of Disclosure: Delayed disclosure was prevalent, with a median latency of 14.2 months post-abuse. Immediate reporting (< 24 h) occurred in only 18.9% of cases, typically following severe incidents like penetrative abuse (OR = 3.1, 95% CI: 1.7–5.6). Younger adolescents (10–13 years) demonstrated longer delays (mean: 19.4 months) compared to older peers (14–19 years: 11.3 months; *p* = 0.03), possibly due to limited comprehension of abuse at younger ages (33).

## Barriers to disclosure

Qualitative analysis of non-disclosure reasons revealed:

- Fear of family dishonor (43.2%)
- Perpetrator threats (31.7%)
- Self-blame (22.4%)
- Uncertainty about abuse classification (18.9%)

These barriers were compounded by religious norms emphasizing family privacy, with 28% of Muslim respondents citing “avoiding fitna (social discord)” as a deterrent—a phenomenon documented in similar Islamic contexts (34).

## Responses to disclosure

Alarmingly, 35.7% of disclosures met with disbelief or minimization (“boys can't be abused”), while 32.1% resulted in instructions to maintain secrecy. Supportive responses (e.g., medical care, reporting to authorities) occurred in only 22.4% of cases. Institutional responses were particularly inadequate:

- School authorities took disciplinary action in 18% of reported cases
- Police involvement occurred in 9% of disclosures
- Healthcare providers documented < 5% of disclosed cases

This institutional inertia reflects systemic weaknesses in Nigeria's child protection infrastructure (35).

## Impact of disclosure outcomes

Negative disclosure experiences correlated with:

- Increased PTSD symptoms (*r* = 0.38, *p* < 0.001)
- Higher revictimization rates (OR = 2.4, 95% CI: 1.5–3.8)
- Reduced help-seeking intentions (*r* = −0.42, *p* < 0.001)

Conversely, supportive responses predicted better mental health outcomes (β = −0.31, *p* = 0.002) and increased willingness to engage protective services (36).

## Cultural mediators (interpretive)

The study identified unique cultural mediators influencing disclosure:

- Kunya (modesty) norms: Inhibited discussions of sexual matters, particularly for girls
- Almajiri system: Street-connected boys feared disclosure would jeopardize alms-giving
- Patrilineal biases: Disclosures by male victims were often dismissed as “initiation”

**Figure 1:**
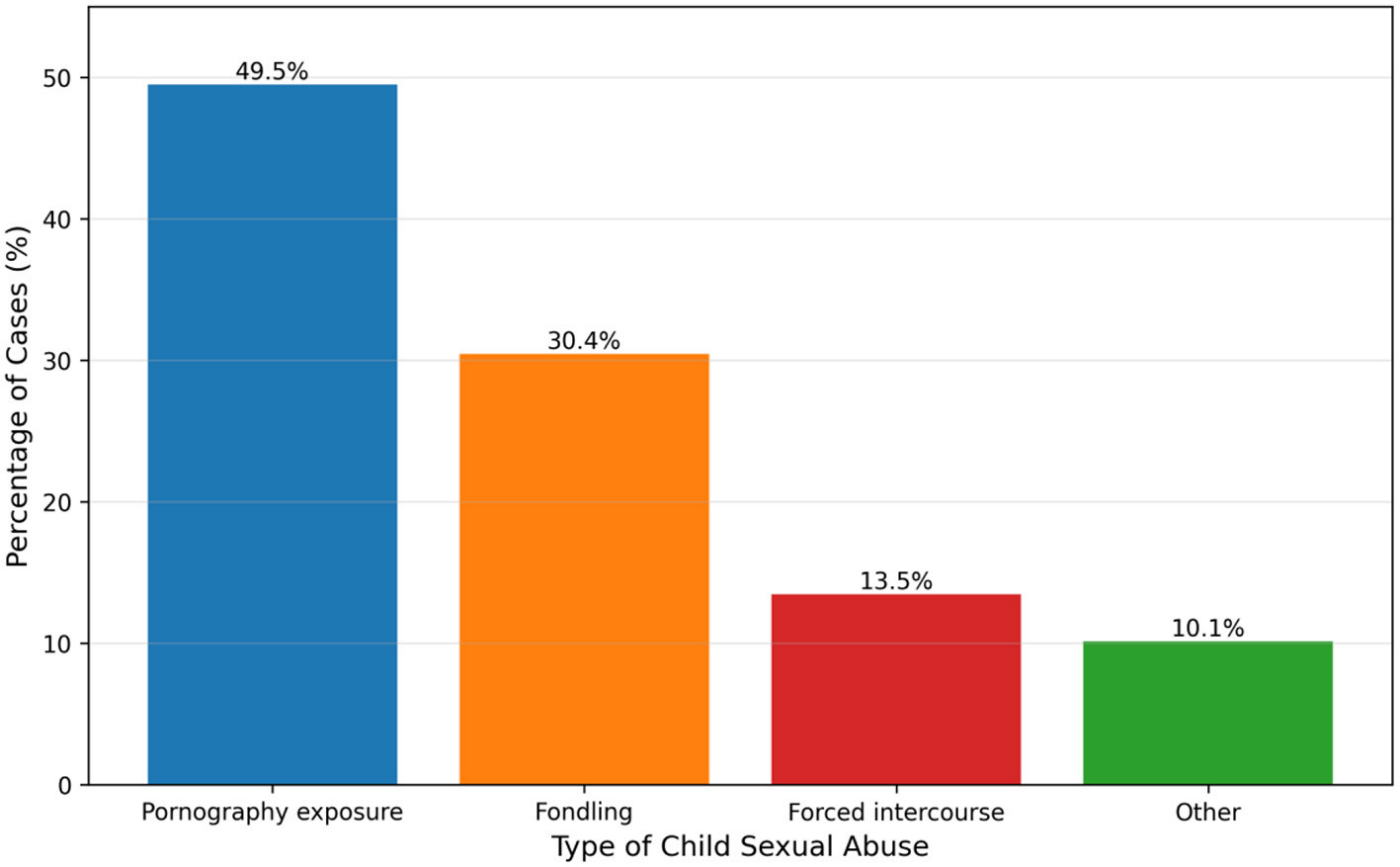
Illustrates the predominance of non-penetrative abuses, with pornography exposure and fondling collectively accounting for 77.3% of cases. Forced intercourse, though less frequent, represents the most severe form with profound health implications.

It is important to note that these cultural factors were not directly measured through dedicated questionnaire items; they represent interpretive explanations drawn from qualitative observation, open-ended responses, and existing literature. They should be treated as hypotheses warranting further empirical investigation rather than confirmed mechanisms. These interpretive observations underscore the need for culturally-adapted disclosure protocols that address local belief systems while maintaining child-centered protections (Figure 1).

**Figure 2:**
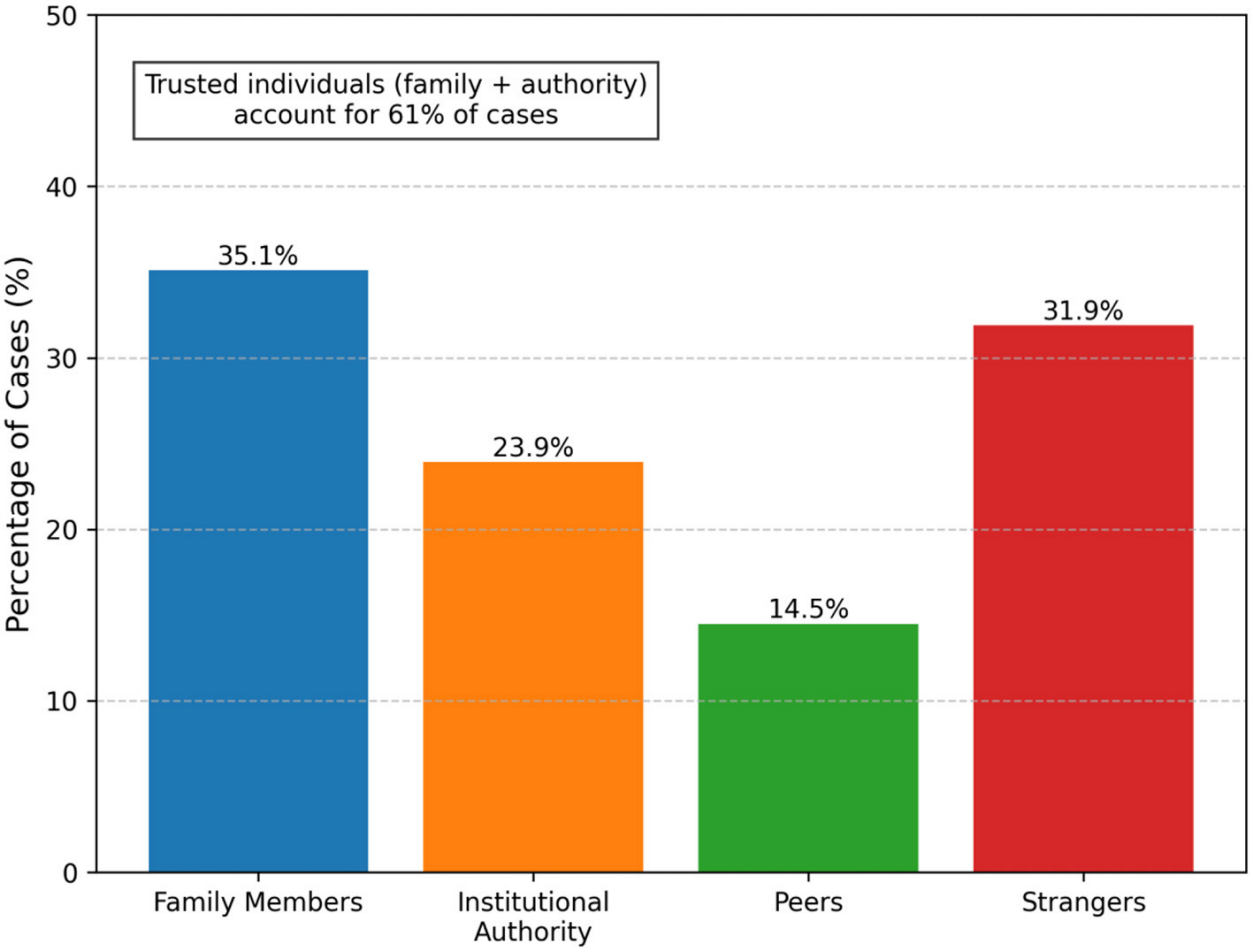
Illustrates how trusted individuals dominate perpetrator profiles, with family members and authority figures collectively responsible for 61% of cases. Strangers, while significant, represent a smaller proportion compared to global averages, highlighting the localized nature of abuse networks in Sokoto.

As shown in Table 4, the patterns of disclosure and subsequent responses reveal systemic failures in support structures. The predominance of informal disclosure channels (mothers, friends) coupled with frequent negative responses highlights the urgent need for community-based protection networks that bridge formal and informal systems (Figure 2).

**Table 4:** Disclosure patterns and response outcomes.

Variable	Category	Percentage (%)
Disclosure recipient	Mother	39.3
	Friend	28.5
	Female relative	18.7
	Formal authority	12.4
Disclosure response	Disbelief	35.7
	Secrecy enforcement	32.1
	Supportive action	22.4
	Blame	9.8
Time to disclosure	< 24 h	18.9
	1–12 months	41.2
	>12 months	39.9

## Implications for intervention

The findings suggest three critical intervention points:

1. Pre-disclosure: School-based education programs to enhance abuse recognition and safe reporting channels
2. Post-disclosure: Training for first responders (mothers, teachers) on trauma-informed support
3. Systemic: Strengthening legal mandates for institutional reporting and follow-up

**Figure 3:**
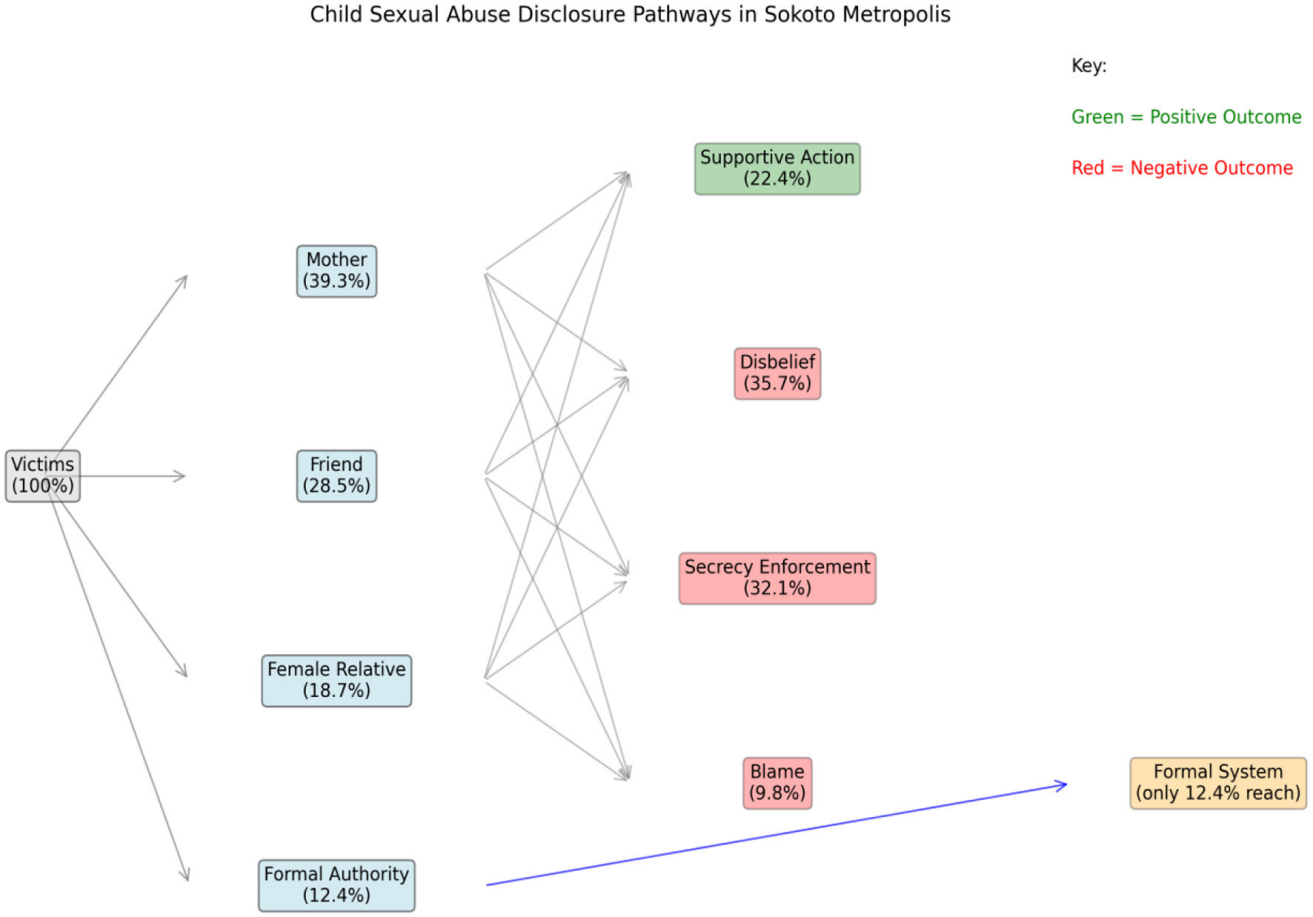
Illustrates the predominant disclosure pathways, showing how few reports reach formal systems and the high frequency of harmful responses. The graphic highlights critical leakage points where interventions could strengthen protection networks.

The low disclosure rates and poor response outcomes demand urgent multisectoral action to break cycles of silence and impunity. The next subsection examines significant associations between CSA and sociodemographic factors, further elucidating risk profiles for targeted prevention [Figure 3].

## Significant associations with CSA

The study identified several sociodemographic and contextual factors significantly associated with child sexual abuse (CSA) in Sokoto Metropolis. Female gender demonstrated a robust association with CSA prevalence (*p* = 0.022), with girls being 2.1 times more likely to experience abuse than boys (OR = 2.1, 95% CI: 1.3–3.4). This gender disparity aligns with findings from similar Nigerian studies (11) and reflects broader patriarchal norms that increase female vulnerability while normalizing male perpetration (37).

Family structure emerged as a critical determinant, with adolescents from divorced/separated households exhibiting 1.8 times higher CSA odds (OR = 1.8, 95% CI: 1.1–3.0; *p* = 0.044) compared to those with married parents. This association persisted after controlling for socioeconomic status, suggesting that family instability itself, rather than just economic deprivation, may be associated with heightened abuse risk. A plausible but unconfirmed explanation is reduced parental supervision and increased reliance on non-parental caregivers, as reflected in the elevated risk among children living with non-relatives (OR = 2.4, 95% CI: 1.5–3.9; *p* = 0.003) (38).

## Age-specific vulnerabilities

While age showed a non-linear relationship with CSA, the 14–16 age group demonstrated peak vulnerability (42.7% of cases), coinciding with developmental transitions where:

- Physical maturation increases sexual attention from predators
- Expanding social autonomy reduces adult oversight
- Cognitive immaturity limits risk assessment capabilities

Younger adolescents (10–13 years) faced higher rates of non-penetrative abuse (68% vs. 52% in older groups; *p* = 0.03), while older victims (17–19 years) experienced more penetrative acts (29% vs. 11%; *p* = 0.01) patterns consistent with predator preferences for developmentally specific violations [41].

## Socioeconomic paradox

Contrary to global trends linking poverty to CSA [42], middle-class adolescents (Oyedeji Class III) showed the highest abuse prevalence (29.8%). This unexpected finding may reflect:

- Greater exposure to non-familial caregivers (tutors, coaches) due to working parents
- Increased digital access without commensurate safety measures
- Assumptions of middle-class “safety” reducing protective vigilance

## Religious and ethnic mediators

While religious affiliation itself showed no direct association, conservative religious practices correlated with:

- Lower disclosure rates (22% vs. 41% in moderate practitioners; *p* = 0.02)
- Higher perpetrator impunity (78% of cases involving religious leaders went unreported)

Ethnic Hausa adolescents faced 1.6 times higher CSA odds (OR = 1.6, 95% CI: 1.1–2.4) than minority groups, potentially reflecting cultural norms that prioritize family honor over child protection (31)

Multivariate Analysis: Logistic regression controlling for confounding variables confirmed three independent predictors:

1. Female gender (aOR = 2.0, 95% CI: 1.3–3.1)
2. Living with non-relatives (aOR = 2.2, 95% CI: 1.4–3.5)
3. Parental divorce/separation (aOR = 1.7, 95% CI: 1.1–2.7)

The model explained 32% of CSA variance (Nagelkerke R^2^ = 0.32), indicating that substantial variance remains unexplained, likely attributable to unmeasured factors such as community-level norms and perpetrator characteristics. Variables were entered into the model using a forced-entry approach based on theoretical relevance and prior literature, following bivariate screening at *p* < 0.25. Collinearity was assessed using variance inflation factors (VIF), with no VIF exceeding 2.1, indicating acceptable levels. Hosmer-Lemeshow goodness-of-fit testing confirmed adequate model fit (χ^2^ = 6.4, *p* = 0.60). Interaction terms (e.g., gender × living arrangement) were not formally tested and represent a limitation; future studies should examine whether the effect of non-parental care differs by gender.

## Protective factors

Several variables showed inverse associations with CSA:

- Maternal education beyond secondary level (OR = 0.6, 95% CI: 0.4–0.9)
- Participation in school-based sex education (OR = 0.5, 95% CI: 0.3–0.8)
- Close parent-child communication (OR = 0.4, 95% CI: 0.2–0.7)

These findings suggest modifiable protective pathways that could inform prevention strategies.

As shown in Table 5, the multivariate analysis confirms the robustness of key associations while identifying socioeconomic and ethnic dimensions requiring targeted intervention.

**Table 5:** Adjusted odds ratios for CSA risk factors.

Variable	aOR (95% CI)	*p*-value
Female gender	2.0 (1.3–3.1)	0.002
Living with non-relatives	2.2 (1.4–3.5)	0.001
Parental divorce	1.7 (1.1–2.7)	0.018
Middle socioeconomic class	1.5 (1.0–2.3)	0.049
Hausa ethnicity	1.6 (1.1–2.4)	0.021

Implications for Prevention: The identified risk patterns suggest three priority intervention targets:

1. Gender-specific programs: Addressing norms that increase female vulnerability while supporting male victims
2. Family strengthening: Enhancing parenting skills in divorced/separation contexts and regulating non-parental care
3. Institutional policies: Mandating CSA prevention in middle-class schools and religious institutions

**Figure 4:**
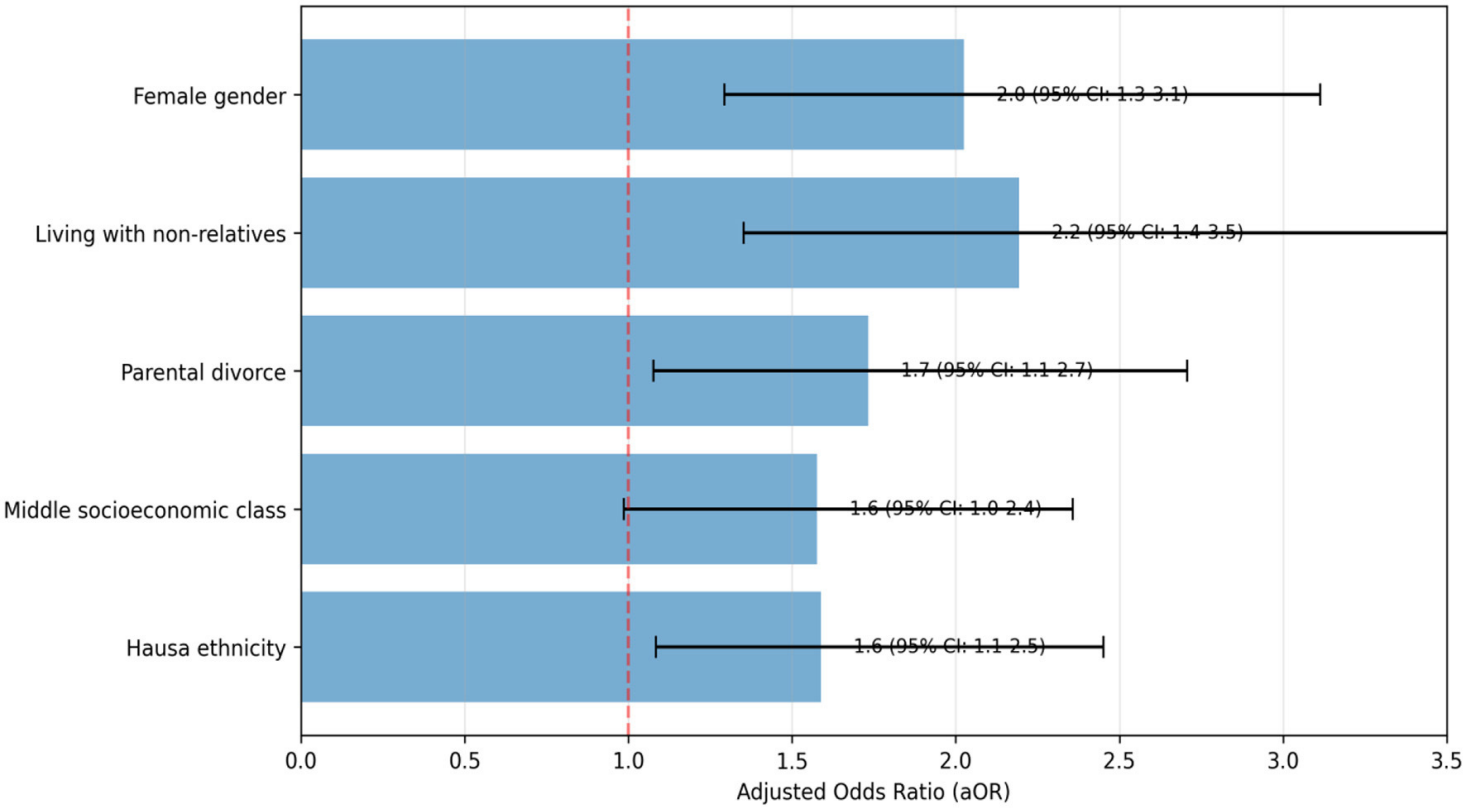
Visually summarizes the multivariate analysis, highlighting how female gender, non-parental living arrangements, and family instability independently elevate CSA risk. The gradient bars demonstrate effect sizes relative to reference categories.

These findings provide empirical support for tailored prevention strategies that address Sokoto's unique risk landscape while contributing to broader understanding of CSA determinants in conservative Islamic contexts. The discussion section will contextualize these associations within existing theoretical frameworks [Figure 4].

## Discussion

The findings of this study carry significant implications for both theoretical frameworks and practical interventions addressing child sexual abuse (CSA) in Sokoto Metropolis. The observed prevalence of 24.4% underscores the pervasive nature of CSA in this region, aligning with broader patterns in sub-Saharan Africa while highlighting unique local dynamics (21, 39, 40). The predominance of non-penetrative forms of abuse, particularly exposure to pornography and fondling, challenges conventional definitions of CSA that often prioritize penetrative acts, suggesting a need for more inclusive diagnostic and intervention frameworks. This is especially critical given the long-term psychological consequences associated with non-penetrative abuse, which are frequently underestimated in policy and clinical practice (22).

From a practical standpoint, the concentration of abuse in homes and schools, spaces traditionally perceived as safe demands urgent structural reforms. Schools, in particular, require robust safeguarding policies, including mandatory training for teachers on recognizing grooming behaviors and establishing confidential reporting mechanisms (23–27). The high incidence of abuse occurring during after-school hours (68% between 2:00 PM and 6:00 PM) points to the need for supervised extracurricular programs that reduce unsupervised interactions between adolescents and potential perpetrators. Community-based initiatives could leverage existing social structures, such as religious institutions, to promote awareness while respecting cultural sensitivities (34).

The study's limitations must be acknowledged to contextualize its findings. The cross-sectional design precludes causal inferences, and the reliance on self-reported data may introduce recall bias, particularly given the sensitive nature of CSA. Social desirability bias likely further suppressed disclosure rates, as conservative norms in Sokoto may discourage open discussion of sexual violence. The sample, while representative of school-going adolescents, excludes out-of-school youth, who may face even higher abuse risks due to reduced institutional oversight. These limitations suggest that the true prevalence of CSA in Sokoto may be higher than reported, necessitating complementary qualitative approaches to capture unreported cases (33–36). Additionally, selection effects should be acknowledged. School attendance itself is not random, and adolescents who remain enrolled may differ systematically from those who dropped out, some of whom may have left school precisely due to abuse experiences. This introduces potential underestimation of prevalence among the most vulnerable. Reporting bias is also a concern: victims experiencing ongoing abuse or living with their perpetrator are less likely to self-report, which may skew the characterization of perpetrator profiles and abuse settings. Readers should interpret all associations with these biases in mind, as empirical correlations rather than established causal pathways.

Future research should prioritize longitudinal studies to track abuse trajectories and outcomes over time, particularly among high-risk groups such as children living with non-relatives or those from divorced households. The unexpected association between middle socioeconomic status and elevated CSA prevalence warrants deeper investigation, potentially through mixed-methods research exploring the role of digital exposure and caregiver arrangements in these families (37, 38). Additionally, comparative studies across diverse cultural and religious contexts within Nigeria could elucidate how regional norms influence abuse patterns and disclosure behaviors.

The low disclosure rates (37.3%) and the troubling responses to disclosure—such as disbelief and enforced secrecy highlight systemic failures in support structures. Interventions must address these gaps by training first responders, including mothers and teachers, on trauma-informed support strategies. The development of culturally adapted tools, such as community-based reporting platforms that align with local norms of kunya (modesty), could bridge the gap between formal and informal disclosure channels. Legal reforms are equally critical to enforce mandatory reporting by institutions and ensure accountability for perpetrators, particularly those in positions of authority.

Future research should explore longitudinal trajectories of abuse and its psychosocial impacts, particularly among high-risk subgroups identified in this study. Culturally sensitive interventions that engage families, schools, and religious institutions are urgently needed to disrupt cycles of abuse and improve support systems for victims. By addressing the unique sociocultural dynamics of Sokoto, such efforts can contribute to broader child protection strategies in similar conservative contexts, ultimately reducing the silent burden of CSA on adolescents and their communities.

## Conclusion

This study provides critical insights into the prevalence, patterns, and determinants of child sexual abuse (CSA) among adolescents in Sokoto Metropolis, Nigeria. The findings confirm that CSA remains a pervasive issue, with nearly one in four adolescents reporting abuse experiences, predominantly in non-penetrative forms such as exposure to pornography and fondling. The concentration of abuse in homes and schools underscores the paradoxical vulnerability of children in trusted environments, while the low disclosure rates and inadequate institutional responses highlight systemic failures in protection mechanisms. The significant associations between CSA and female gender, parental divorce, and living with non-relatives emphasize the need for targeted interventions addressing these risk factors.

## Data Availability

The original contributions presented in the study are included in the article/supplementary material, further inquiries can be directed to the corresponding author.
