# Supplementary material for: Prevalence and pattern of child sexual abuse among secondary adolescents in Sokoto Metropolis, Nigeria: a cross-sectional study
Source: Front Public Health. 2026 Jul 28;14:1805877. doi: 10.3389/fpubh.2026.1805877 (PMC13457647; doi:10.3389/fpubh.2026.1805877)
Supplement: Supplementary file 1 [file Image_1.pdf]

Child Sexual Abuse Among Adolescents in Northern Nigeria

A Longitudinal Follow-up Study in Sokoto Metropolis

308	24.4%	37.3%	80%
Adolescents	CSA Prevalence	Disclosure Rate	Male Perpetrators

FORMS OF ABUSE

- 48% — Exposure to pornography
- 29.3% — Fondling
- 14.7% — Forced sexual intercourse

ABUSE LOCATIONS

- 44% — Home
- 26.7% — School

PERPETRATOR PROFILE

Known to victim • Primarily male (80%)

DISCLOSURE TO:

- Mother (39.3% of disclosures)
- Friends (28.5% of disclosures)

BARRIERS TO DISCLOSURE:

35.7% not believed
32.1% discouraged from reporting

SIGNIFICANT RISK FACTORS

- **Female Gender** — $p = 0.022$
- **Parental Divorce/Separation** — $p = 0.044$
- **Living with Non-relatives** — $p = 0.003$

KEY IMPLICATIONS & RECOMMENDATIONS

- Strengthen child protection policies and enforcement mechanisms
- Implement comprehensive school-based prevention and awareness programs
- Improve community awareness and reduce stigma around disclosure
- Establish accessible, survivor-centered support systems and counseling services

Longitudinal study • School-based sampling • Sokoto Metropolis, Nigeria
